# Increased ribonuclease activity in Solanum tuberosum L. transformed with heterologous genes of apoplastic ribonucleases as a putative approach for production of virus resistant plants

**DOI:** 10.3906/biy-2007-87

**Published:** 2021-02-09

**Authors:** Andrii POTROKHOV, Daria SOSNOVSKA, Olga OVCHARENKO, Irena BUDZANIVSKA, Volodymyr RUDAS, Mykola KUCHUK

**Affiliations:** 1 Institute of Cell Biology and Genetic Engineering NAS Ukraine, Kyiv Ukraine; 2 Virology Department, Educational and Scientific Center, Institute of Biology and Medicine of Taras Shevchenko National University of Kyiv, Kyiv Ukraine

**Keywords:** Extracellular ribonucleases, transgenic potato, plant virus

## Abstract

Viral pathogens seriously decrease the yield of potato (*Solanum tuberosum *L.) –an important agricultural crop. Therefore, there is a demand for potato cultivars resistant to multiple viruses. Ribonucleases (RNases) are supposed to be engaged to antiviral response in plants. Heterologous RNase gene expression provides a tool for production of cultivars with multiple resistance to viruses and viroids. Transgenic potato cultivars Luhivs’ka and Lasynak with heterologous genes *bov* and *ZRNase II* of apoplastic RNases from *Bos taurus* and *Zinnia* *elegans* respectively were obtained via *Agrobacterium*-mediated transformation. The presence of *bov* and *ZRNase II* transgenes was confirmed by PCR analysis. RNase activity was examined by modified Oleshko method. Plants with heterologous ribonuclease genes had higher level of RNase activity compared to nontransgenic ones. Transgenic plants inoculated with *Potato virus Y*, PVY (genus *Potyvirus*, family Potyviridae) demonstrated delayed and less severe symptoms of viral infection. DAS-ELISA confirmed the presence of viral antigens both in transformed and control plants. Visual manifestations of viral infection in transgenic potatoes were milder than in control plants and their development was delayed, but complete elimination of the virus did not occur.

## 1. Introduction

Potato (*Solanum tuberosum *L.) is a crop of the greatest importance that is naturally infected with many types of pathogens. Regarding viral diseases, they could be the reason for potato tubers yield decline, quality and market value deterioration. These diseases cannot be cured and their treatment is not profitable. Potato can be infected with more than 10 harmful viruses (Loebenstein and Gaba, 2012; Karasev and Grey, 2013; Budzanivska et al., 2014) including *Potato virus Y* (PVY) and *Potato virus X* (PVX) that are the most deleterious (El-Absawy et al., 2012; Quenouille et al., 2013; Aseel, 2015). Mixed infections have been observed recently: *Potato virus M* and *Potato virus S *often interfere with PVY (Blanchard et al., 2008). 

PVY is RNA-containing virus. Its genome is represented by a single-stranded, linear (+) RNA molecule (3.1–3.2 × 106 Da), with a poly (A) tail at the 3’-end, and VPg at the 5’-end. Virions are nonenveloped, filamentous, flexible, 730–740 nm long, 11–12 nm in diameter, with an axial channel 2–3 nm in diameter, the symmetry is spiral. PVY is easily transmitted by plant sap through vegetative propagation via tubers or cuttings and by aphids in nonpersistent manner. Virus transmission between tubers is also possible during storage (Hussain et al., 2016). Virus transmission by seeds is known only for two host species, while pollen transmission is not possible at all. The experimental range of hosts includes 495 plant species from 31 families, mostly from *Solanaceae*. PVY exists as a complex of strains that induce a wide variety of symptoms on potato leaves and tubers, leading to significant yield reduction and the loss of tubers’ quality. PVY exhibits a clear ability to evolve through the accumulation of mutations and recombination between different strains, adapting to new varieties of potatoes in different environments (Budzanivska and Polishchuk, 2014). 

Agrotechnical measures aimed to cease the spread of the virus by environmentally friendly insecticides and herbicides are used to control the virus infections (Perring et al., 1999; Boquel et al., 2014; Dupuis et al., 2017). These measures are not always efficacious, additionally, they could result in detrimental influence on the environment. Hence, genetic engineering techniques would be benefitable to agriculture and they would facilitate the process of obtaining virus resistant plant lines. The vast majority of plant viruses have RNA genomes. Ribonucleases (RNases) u1d6b the degradation of u1d6e and thus can participate in plant immunity.

Wounding-induced ribonuclease encoding cDNAs was isolated from *Zinnia elegans* (Ye and Droste, 1996). It was found that expression of *ZRNase II *gene was predominantly induced in response to wounding. ZRNase II mRNA was not detected in unwounded *Zinnia* organs, but the *ZRNase II* gene was induced by 6 h after wounding. This extracellular RNase is transported from cell to apoplast. Hypothetical mechanism of the antiviral effect of extracellular RNase can be based both on the vulnerability of the genomic DNA of the viruses at some stages of their penetration into the plant cell and on the simulation of the programmed cell death: when the integrity of tissues is disturbed, the content of apoplast can penetrate the cytoplasm of damaged cells, in which case active RNases function as “killer” proteins that kill the cell and prevent replication of the genomic RNA of the virus that penetrated it (Kochetov and Shumny, 2017). Destruction of viral RNA by RNases retards the development of the symptoms, mitigate their severity or localize the infection (Trifonova et al., 2007). The number of investigations devoted to transgenic plants with heterologous extracellular RNases is rather limited (Kochetov and Shumny, 2017). This approach provides an opportunity to obtain plants resistant to the broad spectrum of viruses (**S**angaev et al., 2007; Trifonova et al., 2012). The benefits of the approach were proved by transgenic plants of a model species *Nicotiana tabacum* (Sangaev et al., 2011; Sugawara et al., 2016). Transgenic tobacco plants with bovine RNase *bov* gene were resistant to cucumber mosaic virus, whereas transgenic tobacco plants with the *Zinnia elegans* gene *ZRNase II* encoding S-like RNase showed resistance to tobacco mosaic virus. Bovine pancreatic RNase cleaves a single-stranded RNA only after maturation (the deletion of N-end leader peptide during secretion) while unmaturated form of protein is inactive and it exerts its action in alkaline milieu. It is well-studied enzyme: its structure and functional range have been already revealed (Raines, 1998). 

Moreover, recombinant RNase genes of various origin have been recently tested as a tool to defend plants from viruses (Watanabe et al., 1995; Sano et al., 1997; Zhang et al., 2001; Ogawa et al., 2005; Ohno and Ehara Y, 2005; Cao et al., 2013; Milosevic et al., 2013). Transgenic *Triticum aestivum *plants with *E. coli rnc 70* gene, encoding RNase III had a high level of resistance to *Barley mosaic virus* (Zhang et al., 2001) *Zea mays*, that contained the same transgene, had less severe symptoms of viral infection during *Rice black-streaked dwarf virus* infection (Cao et al., 2013). Double-strand RNA specific RNase gene *pac1* from *Schizosaccharamyces pombe *was tested as a potential inducer of multiple virus resistance. Multiviral resistance have been confirmed in transgenic plants with *pac1* gene. It was shown that transgenic tobacco was resistant to *Cucumber mosaic virus* and *Potato virus Y* (Watanabe et al., 1995). *Solanum tuberosum* plants transformed with *pac1 *gene were resistant to *Potato spindle tuber viroid* (Sano et al., 1997). Chrysanthemum (*Dendranthema grandiflora*) containing *pac1* gene had delayed symptoms development induced by infection with *Chrysanthemum stunt viroid* and *Tomato spotted wild virus* (Ogawa et al., 2005). Transgenic soybean overexpressing RNase gene *pac1 *showed increased multiple virus resistance (Yang et al., 2019).

We investigated the influence of heterologous genes *bov* (*Bos taurus *extracellular RNase) and the *ZRNase II *gene (*Zinnia elegans* S-like RNase) on virus resistance of transgenic potato plants.

## 2. Materials and methods

### 2.1. Plant material 

Transgenic potato (*Solanum tuberosum* L) plants cv. Luhivs’ka and Lasynak possessing genes of extracellular ribonucleases of plant (*ZRNase II*) and animal (*bov*) origin were used to determine RNAse activity and resistance to potato virus. The Slov’yanka variety was used in these experiments as additional control, as a variety with relatively high natural virus’s resistance, while Luhivs’ka and Lasunak possess moderate antiviral resistance. 

### 2.2. Genetic transformation and bacterial strain 

**Figure 1:**
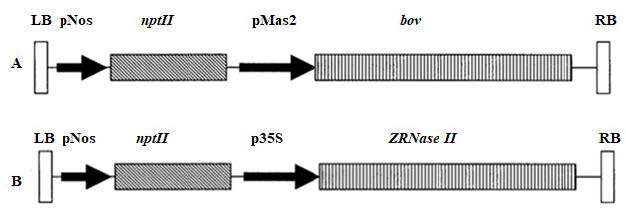
Schematic representation of the T-DNA region of the plasmids рC27bov (A) and pbi-RNS (B) used for potato transformation. LB, left border; pNOS, nopaline synthase promoter; npt II, neomycin phosphotransferase gene; pMas2, constitutive promoter mannopin synthase 2; bov, the cDNA of the Bos taurus pancreatic ribonuclease; 35S, promoter, derived from the cauliflower mosaic virus (CaMV), ZRNase II, S-like RNase gene of Zinnia elegans; RB, right border.

Potato plants (cv. Luhivs’ka and Lasynak) were used for *Agrobacterium*-mediated transformation. Strains of *Agrobacterium tumefaciens* pGV2260 and AGL0 harboring рC27bov vector with gene of pancreatic RNase of *Bos taurus* (*bov*) and pbi-RNS vector with S-like RNase gene of *Zinnia elegans* (*ZRNase II*) respectively were used (Figure 1). T-DNA region of the рC27bov vector contained *bov *gene, the cDNA of the *Bos taurus* pancreatic ribonuclease, under control of constitutive promoter mannopin synthase (pMas2) and *npt II*, neomycin phosphotransferase gene under control of nopaline synthase promoter (pNOS). While T-DNA of pbi-RNS vector included *ZRNase II*, S-like RNase gene of *Zinnia elegans *controlled by p35 S CaMV derived from the cauliflower mosaic virus (CaMV) and *npt II*, neomycin phosphotransferase gene under control of nopaline synthase promoter (pNOS). Neomycin phosphotransferase gene was used as selective marker. 

Genetic transformation and plant regeneration have been conducted as described by Beaujean et al. (1998) with minor modifications in the callus and shoot induction media in transformation protocol. We replaced 0.8 mg/L of zeatin riboside with 0.5 mg/L 6-bensylaminopurine and also added 200 μM of acetosyringone to the callus induction medium. We used zeatin instead of zeatin riboside in shoot induction medium.

### 2.3. PCR assay 

DNA was isolated from putative transgenic plants, positive and negative controls using a standard kit NeoPrep100 DNA plant (Neogene Therapeutics, Inc., City (?), Ukraine) according to the manufacturer’s instructions. The transformation events were confirmed by standard PCR techniques as by Sambrook with PCR MIX 2-R kit (Neogene Therapeutics, Inc.) (Sambrook et al., 1989). The primers and expected size of the PCR fragments are shown in the Table. Gene Ruler DNA Ladder Mix (Fermentas Inc., Waltham, MA, USA) was used as molecular weight markers.

**Table:** Primers used for PCR analyses.

Gene	Primers	Expected size of the PCR fragment, bp.
npt II	5’-CCTGAATGAACTCCAGGACGAGGCA -3’ (F)5’- GCTCTAGATCCAGAGTCCCGCTCAGAAG-3’ (R)	622
bov	5’-ATCATGGCTCTGAAGTTCCC-3’ (F)5’-CCTACACAGTAGCATCAAAG-3’ (R)	450
ZRNAse II	5’-ACACTCGAAGCACACAAACATGAAGA-3’ (F)5’-GAATCTAGAAATTTAGAATGAAGGA-3’ (R)	720

### 2.4. RNase activity detection 

The RNase activity of potato leaf homogenates was measured according to modified Oleshko method (Oleshko et al., 1981). Samples of 200 mg leaf tissue were grounded in 2 mL of distilled water and were centrifuged over 20 min period at 2500 rpm. 

Plant homogenate (0.6 mL), 0.9 mL of total yeast RNA solution (3 mg/mL) in 0.1 M acetate buffer (pH 5.2) and 0.6 mL of 0,1 M acetate buffer (pH 5.2) were mixed and incubated for 30 min at 25 °C. The mixture was placed for 20 min at + 4 °C and further was centrifuged 20 min at 2500 rpm. Optical density measurements were performed on a spectrophotometer Unico 2800 at wavelength 260 nm and 275 nm.

The RNase activity was calculated using the formula:

(11.87 E_260_ –10.4 E_275_) × 2 × 1000/(m:4) 

E_260_ and E_275_ are the optical absorption values at a wavelengths 260 nm and 275 nm, respectively; m is the mass of the plant sample material.

### 2.5. Virus inoculation and detection

*Potato virus Y* was inoculated mechanically into the leaves of potato plants. Sample of PVY was isolated from tomato plants with severe symptoms of viral infection. The presence of viral antigens was confirmed by double antibody sandwich enzyme-linked immunosorbent assays (DAS-ELISA). Plants were grown under 16-h day length with artificial light at 24 °C. 

DAS-ELISA was performed using a standard PVY detection kit (Loewe Biochemica GmbH, Sauerlach, Germany), according to the manufacturer’s instructions.

### 2.6. Statistic evaluation

Analysis of variance was used to calculate the least significant differences based on t-tests at P < 0.05 by Statistica 5.5 and MS Excel 2016 software. Data were analyzed in order to evaluate statistical differences between each transgenic line and corresponding nontransgenic control. Each transgenic line and corresponding nontransgenic (NT) control plants were represented by 6 individual plantlets. The experiments were repeated triple.

## 3. Results

After having conducted *Agrobacterium*-mediated transformation putative transgenic plants have been obtained and were grown on the MS media supplemented with 100 mg/L of kanamycin-sulfate. Three independent putative transgenic potato lines of cv. Lasunak (La 1-3) and 3 of cv. Luhivs’ka (Lu 1-3) were obtained with рC27bov vector. There were regenerated 5 transgenic lines of Lasunak (Las 1-5) cultivar and 8 lines of Luhivs’ka (Luh 1-8) with pbi-RNS vector. Shoots of selected potato lines spontaneously rooted on plant growth regulator free media in the presence of kanamycin.

Putative transformants were PCR tested with specific primers to confirm the presence of heterologous *npt II*, RNase *bov* and *ZRNase II* genes. Nontransgenic plants of Lasunak, Luhivs’ka and Slov’yanka cultivars were also analyzed to ensure the absence of the transgenes. Slov’yanka cultivar is known to have enhanced natural virus resistance, therefore, the plants were analyzed and used as a control in subsequent virological experiments.

**Figure 2:**
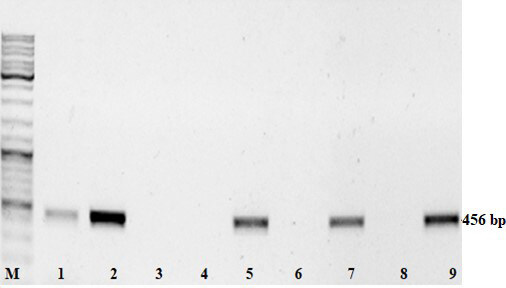
Electrophoregram of PCR products on the bov gene (456 bp). M – molecular weight marker; 1 – Lu-1; 2 – positive control (A. tumefaciens DNA); 3 – negative control without DNA; 4 – nontransformed plant cv. Slov’yanka; 5 – Lu-3; 6 – nontransformed plant cv. Luhivs’ka,; 7 – La-1; 8 – nontransformed plant cv. Lasunak; 9 – La-3.

**Figure 3:**
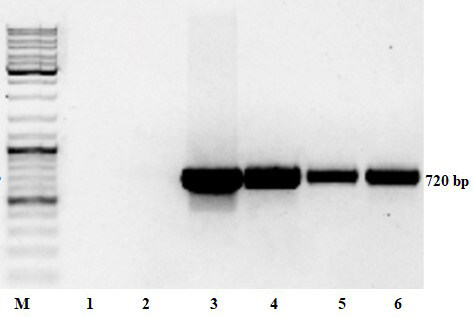
Electrophoregram of PCR products for the ZRNase II gene (720 bp). M – molecular weight marker; 1 – negative control; 2 – Luhivs’ka nontransformed plant; 3 – positive control (A. tumefaciens DNA); 4 – Luh-1; 5 – Luh-3, 6 – Luh-5.

PCR with primers to *bov* gene confirmed the gene of interest integration to genomes of all transformed with рC27bov vector lines, the results are shown in Figure 2. *ZRNase II* gene was present in all lines obtained after pbi-RNS transformation as indicated in Figure 3. All resistant to kanamycin plants revealed *npt II* presence (data not shown). This work is in progress, we showed PCR results of the most vigorous transgenic lines.

**Figure 4:**
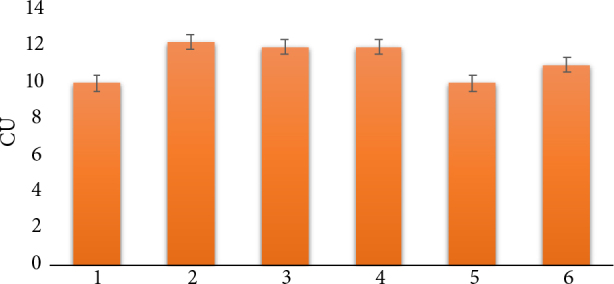
Ribonuclease activity of control and transgenic plants. 1 – Luhivs’ka control; 2 – Luh 1; 3 – Lu 1; 4 – La 3; 5 – Lasunak control; 6 – Slov’yanka control; CU – conventional unit, * significant at P ≤ 0.05.

RNase activity essay was performed to estimate the native RNase level in the utilized in our experiment susceptible cultivars (Luhivs’ka and Lasunak), tolerant cultivar (Slov’yanka) and to compare with the RNase activity in transgenic plants. Two lines with *bov* gene (cv. Luhivs’ka and Lasunak) and a line of Las 1 with *ZRNAse II* gene were examined. Results are represented in Figure 4.

The RNase activity of all tested nontransformed cultivars was on the same level. Transgenic plants have been found to have significantly (P ≤ 0.05) higher levels of RNase activity compared to non-transgenic control. Furthermore, control plants of Slov’yanka had higher levels of RNase activity in comparison to other control plants. The highest value of activity was in transgenic plants of Luhivs’ka cultivar containing the *ZRNase II* gene. RNase activity of the transgenic Lu 1 and La 1lines with the *bov* gene also exceeded the activity of the correspondent nontransformed cultivar.

Plants of different cultivars have been inoculated with PVY. Signs of viral infection were observed after virus inoculation of plants. Symptoms of PVY infection included twisting of the lower leaves, yellow spots on the leaves, necrosis of the stems and the veins, concentric necrosis on the leaves, growth retardation.

**Figure 5:**
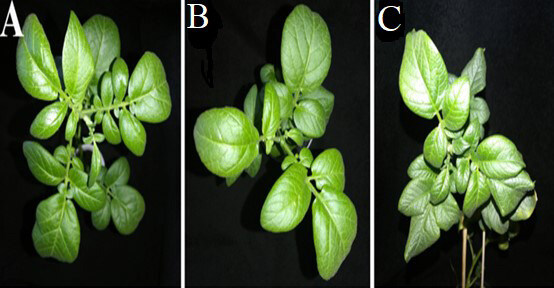
Foliar symptoms caused by Potato virus Y on transgenic and nontransgenic plants of potato cultivar Luhivs’ka: A, plants of the transgenic line with ZRNase II gene, inoculated with buffer; B, plants of the transgenic line with ZRNase II gene, inoculated with the PVY; C, PVY-inoculated nontransgenic Luhivs’ka, control.

The transgenic plants with *ZRNase II* gene inoculated with PVY demonstrated delayed or less severe symptoms of viral infection as indicated in Figure 5. Viral symptoms were completely absent in Luhivs’ka plants with *ZRNase II* gene. We have observed the first developed yellow chlorotic spots on the upper leaves only at 30 days after infection.

**Figure 6:**
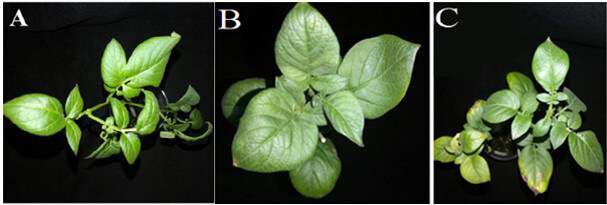
Foliar symptoms caused by Potato virus Y on transgenic and nontransgenic plants of potato cultivar Luhivs’ka: A, plants of the transgenic line with bov gene, inoculated with buffer; B, plant of the transgenic line with bov gene, inoculated with the PVY; C, PVY-inoculated nontransgenic Luhivs’ka, control.

Luhivs’ka with *bov *gene was either completely asymptomatic or had yellow chlorosis and leaf curl as it is shown in Figure 6. In contrast Lasunak with *bov* gene demonstrated yellow spots and necrosis on leaves. 

Nontransgenic plants of cv. Slov’yanka are considered to be quite resistant to viral infection. Nevertheless, there were observed characteristic symptoms after inoculation such as yellow chlorotic spots, small necrosis on the lower leaves, necrosis of stems and veins, concentric necrosis on leaves, growth retardation. 

**Figure 7:**
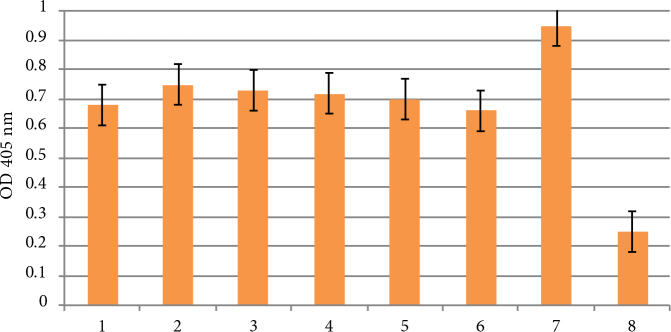
Results of DAS-ELISA: 1 – infected Slov’yanka; 2 – infected Lasunak control; 3 – infected La 3; 4 – infected Luhivs’ka control; 5 – infected Lu 1; 6 – infected Lu 1; 7 – positive control; 8 – negative control, ** significant at P ≤ 0.05 for 7 and 8.

DAS-ELISA have been conducted to verify the presence of the virus in the experimental plants. The results are shown in Figure 7.

According to the DAS-ELISA results, observed levels of virus accumulation varied among cultivars and transgenic lines. The most susceptible cultivar was Lasunak, while Slov’yanka was the most tolerant. There was insignificant difference between nontransformed and transgenic Luhovs’ka with *ZRNase II* gene in the virus concentration.

## 4. Discussion

All plants that were selected from the media with 100 mg/L of kanamycin sulfate were PCR analyzed to determine the presence of heterologous *npt II*, as well as RNase (*bov* and* ZRNase II*) genes. The presence of the transgenes in the transgenic potato plants revealed that the genes were transferred in a cassette.

After inoculation with PVY, nontransgenic plants of three potato cultivars demonstrated different degrees of symptom development. Symptoms of virus infection on non-transformed potato plants varied from the most severe on cv. Lasunak to the mildest on cv. Slov’yanka, thus, indicating variation in cultivar specific resistance. Nontransgenic Slov’yanka plants were used as a reference to evaluate the efficiency of virus infection. Transgenic plants had lower incidence of infection, milder and delayed symptoms in comparison to the nontransformed plants of the same cultivar. Plants of cv. Lasunak with *bov* gene were more susceptible to PVY infection then Luhivs’ka with the same gene demonstrating that basic resistance of cultivar combined with RNase activity plays an important role in total virus resistance. 

DAS-ELISA revealed low level of virus accumulation in Slov’yanka, that was in agreement to the announced characteristics of this cultivar as a resistant one. Nontransgenic plants of Lasunak accumulated the highest level of PVY among all the tested plant groups. There was no significant difference in virus accumulation among control Luhivs’ka and Lasunak and transgenic lines with *bov* genes. In transgenic Luhivs’ka with *ZRNase II* gene, the virus concentration level was the lowest among all the examined groups of plants, that is in consistency with the slight signs of viral infection of this cultivar. In concordance with these results, the accumulation of PVY was significantly inhibited in transgenic plants with *ZRNase II* as was confirmed by DAS-ELISA. Therefore, we can propose that protection provided by expression of *ZRNase II* was more efficient than that of* bov* gene. We attempted to compare our data with the results of similar studies, however, the number of investigations with genes of our interest is limited. Trifonova et al. (2007, 2012) also observed delay in the development of tobacco mosaic virus symptoms in the tobacco plants transformed with *bov *and *ZRNase II* genes.

Introduction of another RNase (dsRNA-specific RNase *pac1*) into *Impatiens walleriana* provided complete resistance to *Tomato spotted wilt virus*, while transgenic tobacco (*Nicotiana tabacum*) lines exhibited either complete resistance or enhanced tolerance to TSWV, evident as lower frequency, prolonged incubation period and milder symptoms in comparison to untransformed plants (Milosevic et al., 2013) The results of Yang et al. (2019) showed that overexpression of heterologous gene coding RNase (*pac1*) in transgenic soybean increased its multiple virus resistance, and thus provided an efficient control strategy against a number of RNA viruses. However, transgenic soybean lines expressing *pac1* also were not entirely immune to viral infections under experimental conditions. The phenomenon was thought to be associated with dose dependent effect of the resistance mediated by PAC1. It had been shown that expression of heterologous RNase could control a wider number of viruses than transgenic strategies based on RNAi-silencing (Yang et al., 2019). The introduction of RNase genes is considered to protect plants not only from a variety of viruses, but also from viroids, such as *Potato spindle tuber viroid* or *Chrysanthemum stunt viroid* that can be digested by *PAC1* gene product (Ogawa et al., 2005). Several viroids are spread worldwide, classified as quarantine pathogens and, hence, there is an urgent need for the development of robust antiviroid strategies. Potato tuber viroid have a wide host range, including crop and ornamental plants, and can cause devastating diseases (Dalakouras et al., 2015).

## 5. Conclusion

Based on the data obtained we came to the following conclusions.

Integration of the genes of interest both with the selective gene to the genome of transgenic plants confirmed the insertion of entire cassette from T-DNA. Thus, transgenic potato plants of cultivars Luhivs’ka and Lasunak with RNase genes of plant and animal origin have been produced.

Transgenic plants with the *ZRNase II* gene demonstrated the highest level of RNase activity and the lowest level of virus accumulation. However, increased RNase activity did not completely prevent the development of viral infection symptoms in transgenic plants. Visual manifestations of viral infection in transgenic potatoes were milder than in control plants, and their development was delayed, but complete elimination of the virus did not occur.

The process of genetic transformation and virus resistance depended on cultivar’s characteristics. Hence, we are planning to expand the experiments by involving more lines of transgenic potatoes of a number of cultivars and infect them with various concentrations of viruses and viroids in the future.

## Contribution of authors

M.V. Kuchuk and I. G. Budzanivska planned the experiments, A. A. Potrochov, D. I. Sosnovska, O.O. Ovcharenko conducted the trials, V.A. Rudas performed A. tumefaciens-mediated transformation, D. I. Sosnovska conducted the virus inoculation assay and DAS-ELISA, A. A. Potrochov, O.O. Ovcharenko and V.A. Rudas drafted the manuscript. All authors participated in the manuscript revision.
